# G6P[8] Rotavirus a Possessing a Wa-like VP3 Gene from a Child with Acute Gastroenteritis Living in the Northwest Amazon Region

**DOI:** 10.3390/pathogens12070956

**Published:** 2023-07-20

**Authors:** Marcia Terezinha Baroni de Moraes, Mauro França da Silva, Yan Cardoso Pimenta, Carina Pacheco Cantelli, Rosane Maria Santos de Assis, Alexandre Madi Fialho, Marina Galvão Bueno, Alberto Ignácio Olivares Olivares, Lennart Svensson, José Paulo Gagliardi Leite, Johan Nordgren

**Affiliations:** 1 Laboratory of Comparative and Environmental Virology, Oswaldo Cruz Institute, Oswaldo Cruz Foundation, Fiocruz, Avenida Brasil, 4365-Manguinhos, Rio de Janeiro 21040-360, Brazil; yancpimenta@gmail.com (Y.C.P.); carina.oliveira@ioc.fiocruz.br (C.P.C.); rmsassis@ioc.fiocruz.br (R.M.S.d.A.); amfialho@ioc.fiocruz.br (A.M.F.); marina.bueno@ioc.fiocruz.br (M.G.B.); jpgleite@ioc.fiocruz.br (J.P.G.L.); 2 Post-Graduate Program in Tropical Medicine, Oswaldo Cruz Institute, Oswaldo Cruz Foundation, Fiocruz, Avenida Brasil, 4365-Manguinhos, Rio de Janeiro 21040-360, Brazil; mauro@bio.fiocruz.br; 3 Technological Coordination, Tetraviral Vaccine, Immunobiological Technology Institute (Biomanguinhos), Oswaldo Cruz Foundation, Fiocruz, Avenida Brasil, 4365-Manguinhos, Rio de Janeiro 21040-360, Brazil; 4 Secretaria Estadual de Saúde de Roraima, SESAU/RR, Rua Madrid, 180-Aeroporto, Boa Vista 69310-043, Brazil; albertoufrr@gmail.com; 5 College of Medicine, State University of Roraima, Avenida Helio Campo, s/n—Centro, Caracaraí, Boa Vista 69360-000, Brazil; 6 Division of Molecular Virology, Department of Clinical and Experimental Medicine, Linköping University, 581 85 Linköping, Sweden; lennart.t.svensson@liu.se (L.S.); johan.nordgren@liu.se (J.N.); 7 Department of Medicine, Kalolinska Institutet, Nobels Väg 6, 171 77 Stockholm, Sweden

**Keywords:** rotavirus, acute gastroenteritis, Amazon region

## Abstract

The introduction of rotavirus A (RVA) vaccines has considerably reduced the RVA-associated mortality among children under 5 years of age worldwide. The ability of RVA to reassort gives rise to different combinations of surface proteins G (glycoprotein, VP7) and P (protease sensitive, VP4) RVA types infecting children. During the epidemiological surveillance of RVA in the Northwest Amazon region, an unusual rotavirus genotype G6P[8] was detected in feces of a 2-year-old child with acute gastroenteritis (AGE) that had been vaccinated with one dose of Rotarix^®^ (RV1). The G6P[8] sample had a DS-1-like constellation with a Wa-like VP3 gene mono-reassortment similar to equine-like G3P[8] that has been frequently detected in Brazil previously. The results presented here reinforce the evolutionary dynamics of RVA and the importance of constant molecular surveillance.

## 1. Introduction

Rotaviruses (RVs) are among the most important gastrointestinal pathogens causing acute gastroenteritis (AGE) in children, young mammals, and birds worldwide [1]. Among children under five years of age, rotavirus group A (RVA) is one of the most common causes of diarrheal deaths [2,3] and the most common cause of AGE, with a particularly heavy burden in developing countries [4,5]. RVA genome [5] is enclosed in a triple-layered capsid comprising 11 segments of double-stranded RNA (dsRNA) that encode six structural proteins (VP1–VP4, VP6 and VP7) and six nonstructural proteins (NSP1–NSP5/6). RVA are classified in a binary system based upon the main neutralization antigens, namely the spike protein (VP4) and the major outer capsid glycoprotein (VP7) [6,7]. In addition, whole genome classification is used to assign genotypes to each gene designated by letters (Gx-P[x]-Ix-Rx-Cx-Mx-Ax-Nx-Tx-Ex-Hx) for each of the 11 genes, VP7-VP4-VP6-VP1-VP2-VP3-NSP1-NSP2-NSP3-NSP4-NSP5/6, respectively [7]. The majority of RVA genomes are assigned to three genotype constellations: Wa-like or genogroup 1 (G/P-I1-R1-C1-M1-A1-N1-T1-E1-H1), DS-1-like or genogroup 2 (G/P-I2-R2-C2-M2-A2-N2-T2-E2-H2) and the less common AU-1-like or genogroup 3 (G/P-I3- R3-C3-M3-A3-N3-T3-E3-H3).

Each constellation is believed to have originated from distinct animal species [6,7], and reassortment between RVA strains within the same genogroup may occur. Four rotavirus vaccines are available on the market and preapproved by the World Health Organization (WHO). In 2006, both monovalent (Rotarix^®^ = RV1) and pentavalent (RotaTeq^®^ = RV5) vaccines were licensed and recommended by the WHO and Pan American Health Organization (PAHO). A total of 17 countries, including Brazil, and 1 territory (Cayman Islands) in Latin America and the Caribbean have introduced one of these vaccines into their National Immunization Program (NIP) [8,9].

The aim of this study was to characterize the DS-1-like constellation of an uncommon G6P[8] genotype detected in a sample from a child with AGE living in the Northwest Amazon region (NWAR). The G6P[8] had a DS-1 like backbone and presented a VP3 gene mono-reassortment Wa-like similar to equine-like G3P[8] detected in Brazil [10,11]. G6 type is the most prevalent genotype detected in cattle worldwide; however, it is rarely found in humans, although a few studies from Africa have detected it in relatively high frequency in certain years [7,12,13,14].

## 2. Results

### 2.1. A G6P[8] RV Was Detected in a Fecal Sample Obtained from a Child Living in the Northwest Amazon Region

The sample was collected from a 2-year-old male child living in the extreme North of Brazil’s limit border with Venezuela in the Amazon rainforest (demarcated indigenous “Yekuana” area of Roraima [RR] state, Alto Alegre municipality). The child was attended in the emergency care unit of “Hospital da Criança de Santo Antonio” (HCSA) in RR [15]. The child had one dose of RV1; feces and saliva collected in parallel were negative when tested using molecular methods for norovirus, sapovirus (feces), human bocavirus and adenovirus [15,16,17]. The RT-qPCR) cycle threshold (Ct) value was 24.82 and the symptoms were abdominal pain, dehydration, fever > 38.5 °C, mucus, and vomit. Blood in the feces was checked at the time of collection and the child was diagnosed with AGE and malnutrition by the pediatrician who accompanied this study. According to phenotyping of the histo blood group antigen (HBGA), the child was secretor and Lewis a-b+ [15].

### 2.2. Sample G6P[8] ID-LVCA: 28,398 Was Initially Genotyped as G9 with Multiplex PCR

G/P genotyping using the Centers for Disease Control and Prevention (CDC), USA, multiplex protocol [10] was initially performed on the ID-LVCA: 28,398 sample. The genotyping showed a band size to VP7 gene on the gel corresponding to the G9/VP7 genotype (data not to shown). Subsequent Sanger nucleotide sequencing and phylogenetic analysis showed it to be a G6/VP7 genotype. To further confirm the G6 G-genotype, the G6aF forward primer [18] and VP7R primer [10] were used, yielding a corresponding band size (data not shown).

### 2.3. Phylogenetic Analysis of the G6P[8] Sample Shows a Constellation Possessing a Wa-like VP3

**Figure 1:**
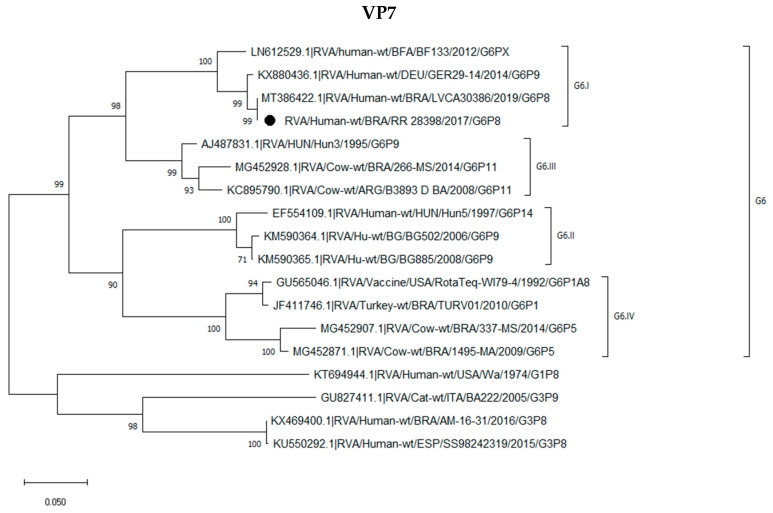

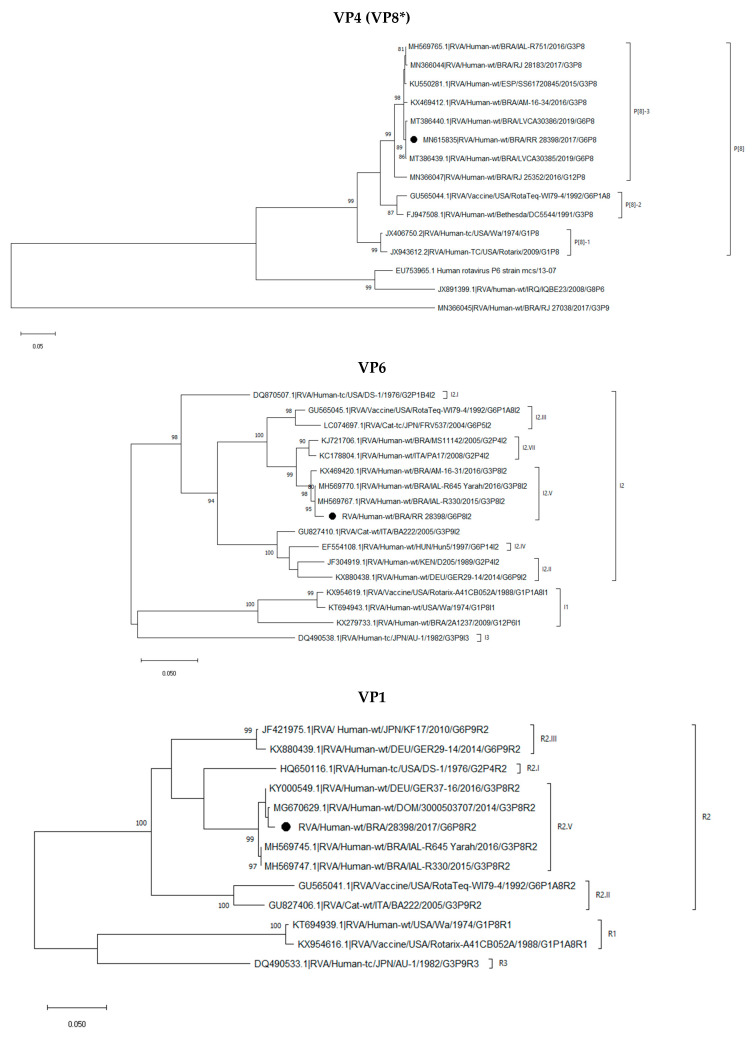

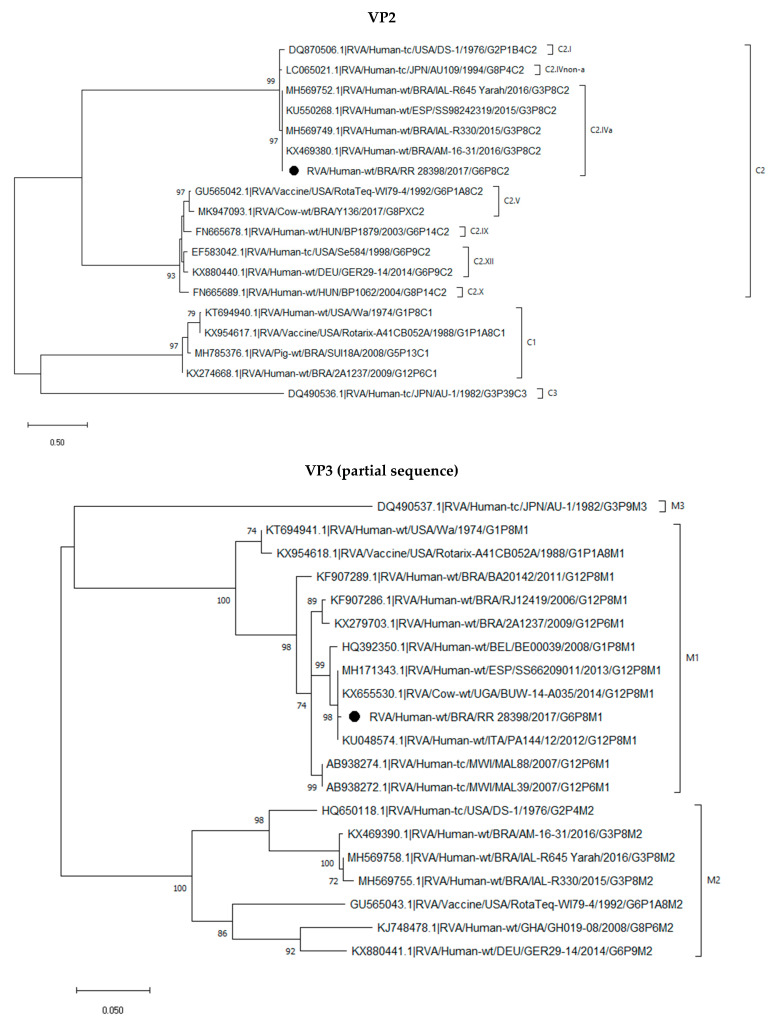

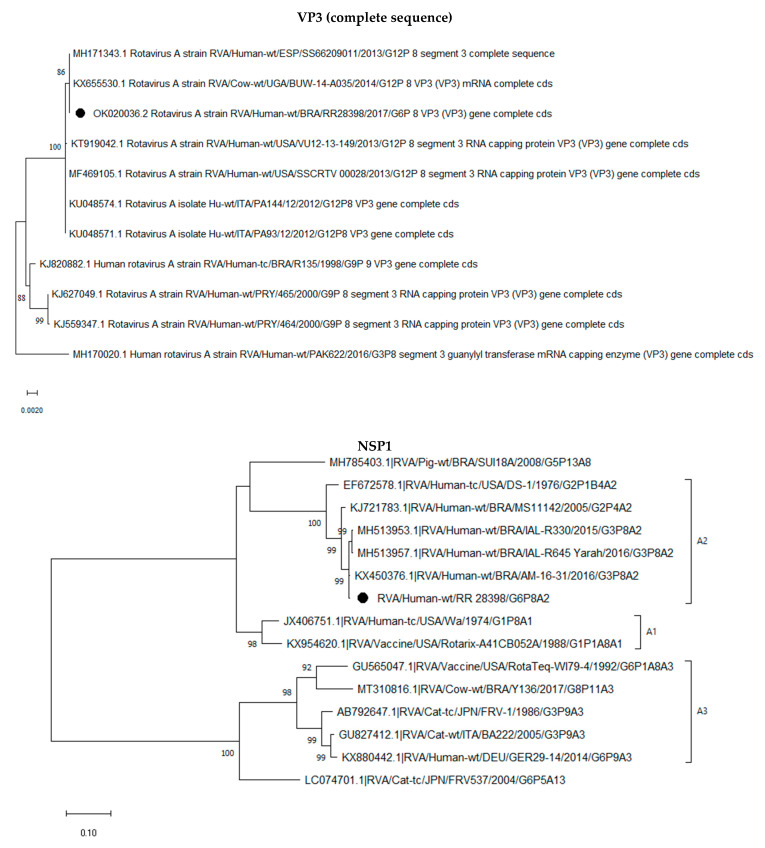

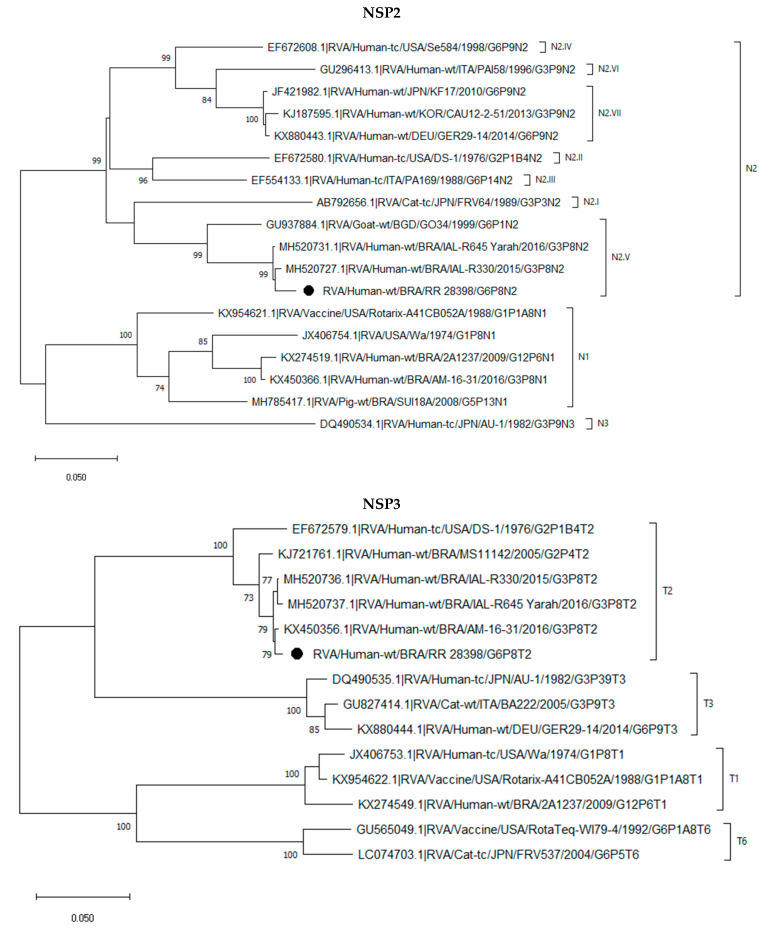

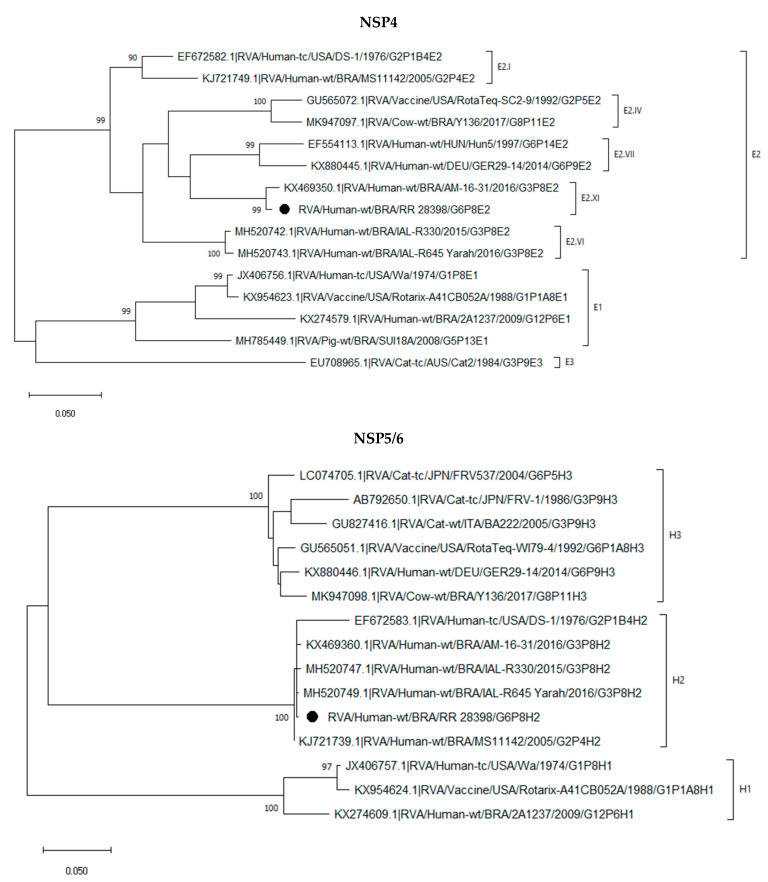
Phylogenetic analyses of the nucleotide (nt) sequences of G6P[8] ID-LVCA: 28,398 sample: (marked with a black-filled circle) structural protein (VPs) 1–3, VP8*, VP6, and VP7) and nonstructural proteins (NSPs) NSP1–NSP5/6 with reference RVA strains from GenBank labeled as follows: number access, species of origin (human, animal or vaccine), RVA group, country, common name, year G and P genotype. Maximum-likelihood phylogenetic trees were constructed with MEGA X software and bootstrap tests (2000 replicates) based on the Tamura three-parameter models. Bootstrap values above 70% are given at branch nodes. Most sequences used for the construction of phylogenetic trees were partial: VP7 (951 nt), VP4 (754 nt), VP6 (1164 nt), VP1 (499 nt), VP2 (555 nt), NSP1 (1194 nt), NSP2 (801 nt), NSP3 (933 nt), NSP4 (519 nt) and NSP5/6 (555 nt) the VP3 gene were analyzed both with partial (574 nt) and complete (2595 nt) sequences. For the analysis of the complete sequence of the VP3 gene, only samples from the RVA M1 group were used. The black dots represent the sample of this study; subgroups are represented by numbers for each letter representing a gene.

To conduct a phylogenetic analysis, we compared the detected G6P[8] (sample ID-LVCA: 28,398) with G6 samples detected in humans and animals. Figure 1 shows the phylogenetics trees generated, one for each of the 11 genes of RVA. The constellation of the ID-LVCA: 28,398 sample was G6-P[8]-I2-R2-C2-M1-A2-N2-T2-E2-H2, similar to the G6P[8] samples that have been detected in Brazil, with the exception of the VP3 gene (Table S1, Supplementary Material). All genes, with the exception of the VP7 and VP3 genes, were similar to the equine-like G3P[8]. The VP7 gene analyses grouped the ID-LVCA: 28,398 sample with the G6.I group related to G6 human samples detected in Brazil, Africa, and Germany. The VP3 gene belonged to genogroup 1/M1 group together with human G12 P[8] and P[6] genotypes, where it exhibited 100% nucleotide identity with the G12 P[8] sample collected from a cow in South Africa [19]. The VP3 gene was subsequently sequenced further (2595 bp), comprising the entire CDS, confirming the 100% match with the sample from a cow in South Africa (GenBank access number KX655530.1). Both partial and complete sequences were used for the construction of the phylogenetic tree of the VP3 gene. The partial sequence was used for a more comprehensive analysis that included more samples with partial nucleotide sequences available in GenBank (Figure 1). The nucleotide sequences of the RV1 and RV5 vaccines genes were from sublines distant from the ID-LVCA: 28,398 sample.

## 3. Discussion

RVA exhibits a vast diversity, and a variety of human strains share genetic and antigenic features with animal origin RVA strains, suggesting that interspecies transmission is an important mechanism of rotavirus evolution and contributes to the diversity of human RVA strains. The G6P[8] sample detected here from a child with AGE living in the NWAR presented G3P[8] DS1-like backbone RVA. This is similar to the G6P[6] strains first detected in a child from Mali and the later emergence with large prevalence detected in west Africa [19]. Unusually, the sample presented a VP3 gene mono-reassortment with some particular characteristics. The G6 (VP7) genotype is unusual in humans, though it frequently occurs in its different P (VP4) combinations in animals including cattle and pigs [20].

In 2013, the new equine-like species RVA G3P[8] DS-1 emerged worldwide [21]. In Brazil, this species appeared in Paraná (South region) in March/2015 and in 2016 it was identified in the North region (Amazon). The rapid dissemination exhibited marked potential to replace circulating Wa-like G3P[8] strains [22]. The analysis of the G3P[8] DS-1 gene constellation identified in the Amazon presented an NSP2 (N1) different from the one then circulating in other regions of Brazil (N2) [23].

The ID-LVCA: 28,398 sample was detected in a child living in the Amazon region and the only RVA sample genotyped in this study was G6 (VP7); it is thus difficult to assess its circulation in the region. G3P[8] and G12P[8] genotypes were the most detected (data not shown), corroborating studies with samples collected during the same collection period in the northern region of Brazil [23,24]. Rare rotavirus genotypes have been observed relatively frequently in the northern region in Brazil [25,26]. Recently, G6P[8] genotypes have been emerging in the Brazilian regions of epidemiological surveillance studies covered by the Regional Rotavirus Reference Laboratory (Southern, Southeastern, and Northeastern) Ministry of Health, Brazil and PAHO (RRRL) [10,11]. The RVA constellation of sample ID-LVCA: 28,398 differs from that of the few that have been deposited in GenBank by exhibiting a different VP3 genotype (M1) Wa-like. The VP3 gene has a 100% match of nucleotide identity with the VP3 gene from a G12 P[8] sample collected from a cow in Africa [18].

The results presented here highlight the continuous evolution of the G6 strains and their potential for global emergence [18,27]. The initial wrong genotyping via the multiplex of sample ID-LVCA: 28,398 may suggest the need for a G6-specific primer in multiplex PCR protocols, the lack of which might lead to an underestimation of the true global frequency.

## 4. Materials and Methods

### 4.1. Sample of This Study and Rotavirus A Detection

The G6P[8] sample (ID-LVCA: 28,398) was collected in September 2017 during an epidemiological investigation study to identify viral etiologic agents causing AGE and virus–host susceptibility in children living in the Amazon rainforest ≤ 5 years old across the span of 1 year (October 2016 to October 2017) [15]. Total viral nucleic acid was obtained from 140 μL of each supernatant of a 10% fecal suspension sample that was subjected to an automatic nucleic acid extraction procedure using a QIAamp^®^ Viral RNA Mini kit (QIAGEN, Redwood City, CA, USA) and a QIAcube^®^ automated system (QIAGEN), according to the manufacturer’s instructions, with a final elute sample volume of 60 μL. The total viral nucleic acids isolated were immediately stored at −80 °C until molecular analysis. Monoplex reverse transcription-quantitative polymerase chain reaction (RT-qPCR) was performed for the detection of RVA [15]. The near-complete nucleotide sequence of the VP3 gene, containing the complete coding DNA sequence (CDS), of the G6P[8] sample was obtained via Sanger sequencing (2595 bp), with primers as previously described [28,29,30].

### 4.2. G/P Rotavirus A Genotyping

The fecal sample was subjected to G and P genotyping in the RRRL using a one-step multiplex RT-PCR as described [10]. Conserved forward primers VP7uF or VP4uF and reverse primers specific for G types G1, G2, G3, G4, G9 and G12, or P types P[4], P[6], P[8], P[9] and P[10] were used as recommended by the CDC, Atlanda, GA, USA. The G and P genotypes were assigned based on different amplicon sizes (base pairs (bp)) using agarose gel analysis. Additionally, to confirm the G6/Vp7 genotype, a single G/RVA genotyping was performed with a previously described specific G6aF (forward) [18] and VP7R [10] primers using the same cycling conditions [10].

### 4.3. Full Genotyping of Rotavirus A ID-LVCA: 28,398 Sample

The processing of the fecal sample, RNA extraction and RVA detection via RT-qPCR has been previously described [15]. cDNA synthesis was performed using random primer and illustra™ Ready-to-go RT-PCR beads (GE HealthCare, Uppland, Uppsala, Sweden), according to the manufacturer’s instructions, except for the VP3 gene, where a VP3 reverse primer was used (Table S2, Supplementary Material). The cDNA was subsequently used for single rounds PCR reactions in a final volume of 25 µL using iTaq (Bio Rad Laboratories, Solna, Stockholm, Sweden) according to the manufacturer’s instructions. The cycling programs were initial denaturation at 94 °C for 10 min, followed by 40 cycles of 94 °C for 30 s, 45 °C to 53 °C for 30 s, 72 °C for 1 to 2 min, and a final extension at 72 °C for 7 min. For the Sanger sequencing, the amplicons were sent to Macrogen Europe B.V. Company (North Holland, Amsterdam, The Netherlands) with the same PCR primers used for PCR amplicons (Table S2, Supplementary Material).

### 4.4. Phylogenetic Trees and RVA Genome Designation

The chromatograms of the RVA gene constellation of sample ID-LVCA: 28,398 were analyzed using the free tracer viewer Chromas 2.4 (Technelysium Pty Ltd., South Brisbane, QL, Australia). RVA nucleotide alignment was carried out using the Mega Molecular Evolutionary Genetic Analysis Version 11 software and compared with reference nucleotide sequences of RVA available in the GenBank database of the National Center for Biotechnology Information (NCBI). Genome designation (Table S1, Supplementary Material) was carried out using the Rotavirus A Genotyping Tool Version 0.1 (https://www.rivm.nl/mpf/typingtool/rotavirusa/how-to-use, accessed on 14 July 2023).

## 5. Conclusions

In this study, we described an unusual constellation of a G6P[8] genotype, containing DS-1like backbone with a Wa-like VP3 (M1) gene. The results are important in the context of the genetic variability and evolution of RVA strains, which may be important for vaccine efficacy.

## Data Availability

The representative gene sequences of RVA G6P[8] sample ID-LVCA: 28,398 obtained in the current study were submitted to GenBank under the access numbers OK020034 to OK020036 (VPs 1–3), MN615835 (VP8*), OK020037 (VP6) and MN615836 (VP7); OK020029- OK020033 (NSP1-NSP5/6).

## Supplementary Materials

The following supporting information can be downloaded at: https://www.mdpi.com/article/10.3390/pathogens12070956/s1, Table S1: Genotype constellation of human Brazilian rotavirus strains G6P[8] and G3P[8], G3P[8] (Spain and Japan) and bovine G12P[8]. The G6P[8] strain detected in this study (sample ID-LVCA: 28,398) is shown in bold; Table S2: Primers used for PCR amplification of the different genes of the G6P[8] strain detected in this.
