# Supplementary material for: G6P[8] Rotavirus a Possessing a Wa-like VP3 Gene from a Child with Acute Gastroenteritis Living in the Northwest Amazon Region
Source: Pathogens. 2023 Jul 20;12(7):956. doi: 10.3390/pathogens12070956 (PMC10385053; doi:10.3390/pathogens12070956)
Supplement: Supplementary file 1 [file pathogens-12-00956-s001.zip › pathogens-2402318-supplementary.pdf]

Supplementary Materials

Table S1. Genotype constellation of human Brazilian rotavirus strains G6P[8] and G3P[8], G3P[8] (Spain and Japan) and bovine G12P[8]. The G6P[8] strain detected in this study (sample ID-LVCA: 28398) is shown in bold.

Genogroup	Rotavirus Atrain	Ref.	Origin	Genotypes										
				VP7	VP4	VP6	VP1	VP2	VP3	NSP1	NSP2	NSP3	NSP4	NSP5/6
DS-like	RVA/Human-wt/BRA/LVCA_30388/2019/G6P[8	11	Human	G6	P[8]	I2	R2	C2	M2	A2	N2	T2	E2	H2
	RVA/Human-wt/BRA/LVCA_30390/2019/G6P[8]	11	Human	G6	P[8]	I2	R2	C2	M2	A2	N2	T2	E2	H2
	RVA/Human-wt/BRA/LVCA_30391/2019/G6P[8]	11	Human	G6	P[8]	I2	R2	C2	M2	A2	N2	T2	E2	H2
	RVA/Human-wt/BRA/AM-16-34/2016/G3P[8]	22	Human	G3	P[8]	I2	R2	C2	M2	A2	N1	T2	E2	H2
	**RVA/Human-wt/BRA/RR_28398/2017/G6P[8]**	**This study**	**Human**	**G6**	**P[8]**	**I2**	**R2**	**C2**	**M1**	**A2**	**N2**	**T2**	**E2**	**H2**
	RVA/Human-wt/ESP/SS61720845/2015/G3P[8]	21	Human	G3	P[8]	I2	R2	C2	M2	A2	N2	T2	E2	H2
	RVA/Human-wt/DEU/GER29-14/2014/G6P[9]	27	Human	G6	P[9]	I2	R2	C2	M2	A3	N2	T3	E2	H3
Wa-like	RVA/Cow-wt/UGA/BUW-14-A035/2014/G12P[8]	19	Bovine	G12	P[8]	I1	R1	C1	M1	A1	N1	T1	E1	H1
AU-1-like	RVA/Human-tc/JPN/AU-1/1982/G3P3[9]	30	Human	G3	P[9]	I3	R3	C3	M3	A3	N3	T3	E3	H3

Table S2. Primers used for PCR amplification of the different genes of the G6P[8] strain detected in this.

Gene/Name [1]	Primer Nucleotide Sequence (5′-3′)	Reference [2]
**VP7** -Vp7G6a (F)	AGA CGG TGT AAA TCA TAA GT	[18]
**VP7** -VP7 R	AACTTGCCACCATTTTTTCC	[10]
**VP4** -VP4 F	TATGCTCCA GTN AATTGG	[10]
**VP4** -VP4R	ATTGCATTTCTTTCCATAATG	[10]
**VP6 -** GEN-VP6F	GGCTTTWAAACGAAGTCTTC	[6]
**VP6 -** GEN-VP6R	GGTCACATCCTCTCACT	[6]
**VP1** -VP1-Uf	TCAGGAATAGCTGATGAAATTGC	[29]
**VP1** -VP1-Ur	AATAATTGATAACATCCATAATTA	[29]
**VP2** -VP2-Uf	GCTATTAAAGGCTCAATGGCGTAC	[29]
**VP2** -VP2-Ur	GGATGTAGAATTGATGGATAATTG	[29]
**VP3 -** G6VP3FelF	CTAATCTCACTACGCATAATATAC	This study
**VP3 -** G6VP3FelR	CTATCCAATGGATCCCACGTCTCA	This study
**NSP1** -GEN_NSP1F	GGCTTTTTTTTATGAAAAGTCTTG	[6]
**NSP1** -GEN_NSP1R	GGTCACATTTTATGCTGCC	[6]
**NSP2** -GEN_NSP2F	GGCTTTTAAAGCGTCTCAG	[6]
**NSP2** -GEN_NSP2R	GGTCACATAAGCGCTTTC	[6]
**NSP3** -MAX-NSP3F	GGCTTTTAATGCTTTTCAGTG	[29]
**NSP3** -MAX-NSP3R	GGTCACATAACGCCCCTATAG	[29]
**NSP4** -GEN_NSP4F	GGCTTTTAAAAGTTCTGTTCC	[6]
**NSP4** -GEN_NSP4R	GGWYACRYTAAGACCRTTCC	[6]
NSP5/6 -MAX-11F	GGC TTT AAA GCG CTA CAG TGA	[6]
NSP5/6 -MAX-11R	GGT CAC AAA ACG GGA GTG GGG	[6]

[1] F = Forward primer; R = Reverse primer; [2] = consider the cited references of the paper.
